# Emission of Carbon Dioxide Influenced by Different Water Levels from Soil Incubated Organic Residues

**DOI:** 10.1155/2013/638582

**Published:** 2013-09-12

**Authors:** M. B. Hossain, A. B. Puteh

**Affiliations:** ^1^Soil Science Division, Bangladesh Institute of Nuclear Agriculture (BINA), Bangladesh Agricultural University (BAU) Campus, P.O. Box 4, Mymensingh 2200, Bangladesh; ^2^Department of Crop Science, Faculty of Agriculture, University Putra Malaysia, 43400 Serdang, Selangor, Malaysia

## Abstract

We studied the influence of different organic residues and water levels on decomposition rate and carbon sequestration in soil. Organic residues (rice straw, rice root, cow dung, and poultry litter) including control were tested under moistened and flooding systems. An experiment was laid out as a complete randomized design at 25°C for 120 days. Higher CO_2_-C (265.45 mg) emission was observed in moistened condition than in flooding condition from 7 to 120 days. Among the organic residues, poultry litter produced the highest CO_2_-C emission. Poultry litter with soil mixture increased 121% cumulative CO_2_-C compared to control. On average, about 38% of added poultry litter C was mineralized to CO_2_-C. Maximum CO_2_-C was found in 7 days after incubation and thereafter CO_2_-C emission was decreased with the increase of time. Control produced the lowest CO_2_-C (158.23 mg). Poultry litter produced maximum cumulative CO_2_-C (349.91 mg). Maximum organic carbon was obtained in cow dung which followed by other organic residues. Organic residues along with flooding condition decreased cumulative CO_2_-C, *k* value and increased organic C in soil. Maximum *k* value was found in poultry litter and control. Incorpored rice straw increased organic carbon and decreased *k* value (0.003 g d^−1^) in soil. In conclusion, rice straw and poultry litter were suitable for improving soil carbon.

## 1. Introduction

Soil organic carbon (SOC) is a key indicator of fertility and quality of the arable fields [1]. It has crucial role in nutrient cycling, improving soil physical, chemical, and biological properties; crop productivity; and reducing greenhouse gases (GHGs) [2]. Before Green Revolution, crop residues were traditionally used for animal feed and returned to soil as organic manures in Bangladesh. However, this practice of straw addition to arable fields has declined due to ever increasing population and indiscriminate use of inorganic fertilizers. Moreover, crop straw removal, less manure addition, and other agronomic practices with low organic carbon returns to arable soils have depleted SOC contents [3]. Decline in SOC is an increasing scientific issue in Bangladesh, threatening soil quality and environmental health. Good soil fertility and environmental health are needed to feed our overgrowing people through increasing crop production.

**Figure 1:**
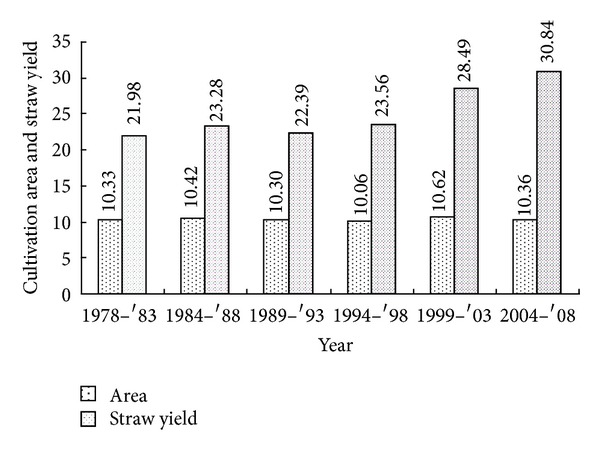
Yearwise distribution of rice growing area (ha × 10^6^) and rice straw yield (mt × 10^6^) in Bangladesh.

Rice-rice (RR) is a major cropping system in subcontinent covering about 10.36 million ha in Bangladesh and another 46.94 million ha in Bhutan, India, Nepal, Pakistan and Sri Lanka [4]. Most of the farmers are using high dose of chemical fertilizer especially urea for the production of rice. As a result, imbalanced nutrition is the factor responsible for the observed declining yield of rice. In Bangladesh, rice growing areas are more or less similar trend from 1978 to 2008, but the production of rice straw showed increasing trend from 1978 to 2008 due to cultivation of high yielding varieties (Figure 1). 

On the other hand, for each kilogram (kg) of feed consumed chicken produces one kg fresh manure with variable water content. So, huge amount of poultry litter is produced from about 98 million poultry in Bangladesh, creating environmental problems in some locations. Cow dung is unavailable in Bangladesh due to use of fuel purpose. Normally rice root is left over after crop harvest but the information should be gathered for better understanding to sustain soil fertility during crop production. If poultry excreta and rice straw are not used as fuel or fodder, these can be a very good source of organic matter in the crop fields. So, the use of cost effective and easily available organic residues in rice-rice cropping system could be an affordable soil management practices for sustainable crop production. Rice straw and poultry litter composting as a method of producing organic fertilizer has limited popularity with farmers for two reasons. It requires extra labour and the compost normally takes 3-4 months to mature. In this regard, rice straw and poultry litter incorporations are alternative management in place of burning or compost before land preparation. But straw incorporation into the soil contributes to reducing condition in the rice field and possible negative impacts on rice growth, because straw incorporation into the soils reduces oxygen and increases toxic carbon compounds. Severe oxygen depletion in flooded soil is related to disease occurrence. Rice straw incorporation into the soil at the rate of 5.0 t ha^−1^ did not affect early rice growth stage or rice yield [5]. However, influence of incorporated rice straw on CO_2_-C evolution and accumulation of soil organic carbon during decomposition are still not clear. Water level is one of the most important factors for decomposition of organic residues in soil. A number of studies has shown that soil moisture could greatly enhance organic residues decomposition and CO_2_ flux [6] or reduce it [7]. Extensive experimentation should be conducted to gain better understanding of decomposition constant rate (*k*) for selecting effective carbon sequester in soil and reduce environmental pollution under different water levels. In this regard, the information on the use of organic residues along with different levels of water is, however, meager on decomposition constant rate. Keeping in view the situation of nominal quantity of organic matter status and low fertility in soils of Bangladesh, an incubation study was conducted to find out the effective carbon sequester such as rice straw and poultry litter in soil and minimized GHGs compared with cow dung, rice root, and control in combination with different water levels on decomposition constant rate by cumulative CO_2_-C evolution and carbon sequestration in soil.

## 2. Materials and Methods

An incubation experiment was conducted at the Soil Science Division under the Bangladesh Institute of Nuclear Agriculture (BINA), Mymensingh, Bangladesh (24°43′43′′N, 90°25′77′′E, 82.296 m above mean sea level) located in Bangladesh. The area receives an average of 2666 mm of annual rainfall, about 76% of which occurs from July to September. The mean minimum and maximum temperatures during the rice growing wet season (July–October) were 26 and 32°C, whereas during the dry season (November–April), they were 17 and 26°C, respectively. The climate of this region is subtropical, semiarid. The soil used in this study was collected in 2012 from the Bangladesh Agricultural University farm, Mymensingh, Bangladesh. Soil samples were collected from surface horizon (0–15 cm) using soil auger. The soil was air dried ground, sieved through 2 mm sieve, and kept in plastic bags for physicochemical analysis. The texture of soil was sandy loam with pH 7.3, 9.2 g kg^−1^ organic C, 0.01% 0.5 mol L^−1^ NaHCO_3_-extractable P, and 0.003% of 1 mol L^−1^ NH_4_OAc-extractable K and C : N ratio 11.5 (Table 1).

Bulk density of 0–15 cm layer measured using cores, and it was 1.28 g/cc. Rice straw and rice root were collected from rice fields after grain harvest. Then it was washed with distilled water and dried at 70°C in laboratory. Rice straw, rice root, cow dung, and poultry litter C were 48.90, 42.20, 17.43, and 47.41%; and total NPK contents were 0.63, 0.40, 1.04, and 1.00; 0.08, 0.29, 0.82, and 0.69%; and 2.35, 0.34, 0.68, and 0.95%, respectively, (Table 1). C : N ratio was 77.61, 105.5, 16.75 and 47.41 in rice straw, rice root, cow dung, and poultry litter, respectively. The straw was cut into small pieces (<1 cm), ground, and mixed with soil samples for incubation. The experiment was setup using complete randomized design with 10 treatments, replicated five times (each 5th replication as control). Rice straw, rice root, cow dung, and poultry litter including control where no use of organic residues were tested under two water levels, that is, moistened condition : field capacity (MC) and flooding condition −2 cm water level (FC) systems. Organic residues were thoroughly mixed with soil then transferred into air tight PVC pots for an equivalent of 200 g soil to 0.25 g C per pot. Samples were wetted slowly with calculated amount of deionized water to maintain designed water level. The pots were then incubated at the constant temperature of 25°C.

The pots filled soil with rice straw, rice root, cow dung, and poultry litter and 50 mL beaker containing 25 mL of 0.05 N NaOH solutions were placed on soil surface inside the pot to absorb carbon dioxide. Pots were covered with polyethylene sheets and incubated in the darkness at 25°C for 120 days. Excess NaOH was titrated with 0.05 N HCl after precipitating carbonates with BaCl_2_ using phenolphthalein as indicator and subtracted from the amount titrated in absolute control where no soil and organic residues were used. All the pots were taken out and opened periodically, aerated for a few minutes, and soil water content was checked and adjusted by weighing then adding distilled water to maintain water levels. The CO_2_-C evolved was measured at 7, 14, 21, 28, 35, 42, 49, 56, 63, 70, 77, 84, 91, 98, 105, 112, and 120th day of incubation. 

The amount of CO_2_-C was calculated by using the following formula:

(1) $$\begin{array}{c} \frac{\text{mg}\;\text{evolved}\;{\text{CO}}_{2}}{\text{day}}=\frac{\left(T_{2}-T_{1}\right)M\times 22}{t}, \end{array}$$

where, *T*_1_ = amount of HCl used to neutralize NaOH, *T*_2_ = *T*_1_ + amount of HCl used to dissolve precipited BaCO_3_, *M* = molarity of HCl, 22 = 22 mg CO_2_/1 mL 1 M HCL, and *t* = time in days. The CO_2_-C in the control treatment was subtracted from the calculated value for CO_2_-C release. At the end of incubation, soil samples were analyzed for organic carbon (SOC) content in soil. SOC concentration was determined using dichromate H_2_SO_4_-K_2_Cr_2_O_7_ wet oxidation method of Walkley and Black. 

A simple model was used to predict the rate of carbon change in soil is shown in the following equation [8]:

(2) $$\begin{array}{c} C_{t}=C_{0}\;\left(1-e^{-kt}\right), \end{array}$$

where *k* is the decomposition constant, *C*_0_ is the potentially mineralisable carbon, and *C*_*t*_ is the carbon mineralization in time *t*. The recorded data were analyzed using two-way analysis of variance (ANOVA) by the statistical package MSTA-c program and means following least significant difference (LSD) test at 5% level of probability for interpretation of results.

## 3. Results and Discussion

**Figure 2:**
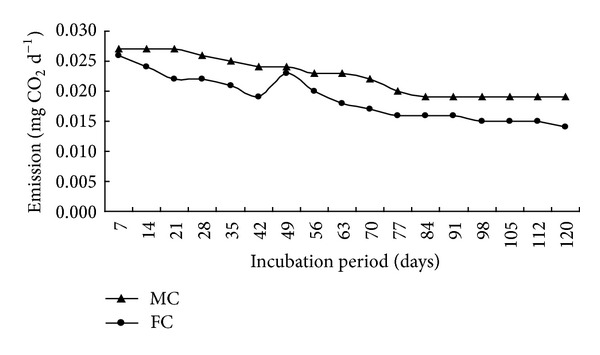
Rate of decomposition at different water levels.

Effect of water levels on carbon dioxide emission rate and cumulative CO_2_-C results are presented (Figures 2 and 4). 

Water level such as moistened condition hastened the CO_2_-C evolution during decomposition of organic residues. Carbon dioxide emission results were statistically significant at all the studied durations except at 7th and 49th day. Higher carbon dioxide emission was observed in moistened condition than in flooding condition. Maximum carbon dioxide emission (0.027 and 0.026 mg d^−1 ^g^−1^ soil) was found at 7 days after incubation in moistened and flooding conditions, respectively, then it decreased with the increase of time except at the 49th day after incubation. The CO_2_-C loss/SOC average ratio was 0.071 and 0.056 in moistened and flooding conditions, respectively. Similar trend was found in cumulative carbon dioxide emission from moistened and continuous flooding systems. Maximum cumulative carbon dioxide emission was observed 265.45 and 218.28 mg CO_2_-C in moistened and flooding conditions at 120 days after incubation. Total input and output carbon, uncounted carbon and carbon degradation constant results are presented (Table 3). Soil and organic residue contributed 0.92 and 0.20 g C per pot, respectively. Higher carbon (0.072 g) emission was found in moistened condition, and the lower carbon (0.058 g) emission was observed in continuous flooding condition.

However, in treatments with moistened condition increased 21.61% CO_2_-C emission over flooding condition. Maximum residual carbon (1.032 g) was achieved in flooding condition, and the lowest carbon (1.012 g) was found in moistened condition. Higher uncounted carbon balance (0.036 g) was found in moistened condition. Maximum carbon degradation rate (0.007 g C d^−1^) was obtained from moistened condition due to higher oxidation process. Moistened condition enhanced the oxidation process of organic residues during incubation periods. Carbon degradation rate was lower in anaerobic condition than in aerobic condition. Organic residue decomposers need oxygen for their respiration process during decomposition. In this regard, flooding condition creates anaerobic status in soil. The cumulative CO_2_-C production significantly decreased with increasing moisture levels (moistened > flooding system) for the entire incubation period. CO_2_-C emission was inclined towards fairly higher moisture levels. This finding may rule out negative influence of flooding condition on microbial activity due to anaerobic conditions. Researchers reported that mineralization of organic residues highly depended on water levels of soil in incubation [6]. Other researchers claimed that CO_2_-C evolution increased up to 60–80% while, suppressed at 100% moisture level. As a result, flooding condition yielded more SOC than moistened condition [7].

**Figure 3:**
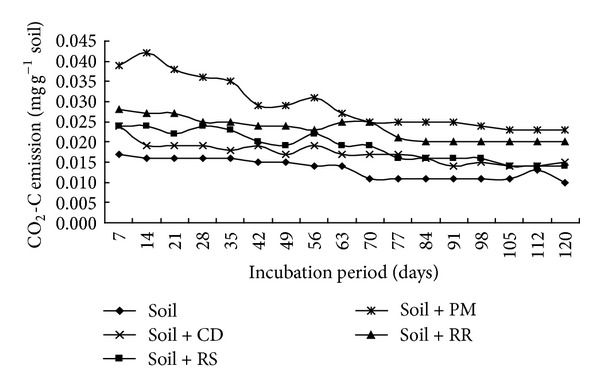
Rate of decomposition from different organic residues.

**Figure 4:**
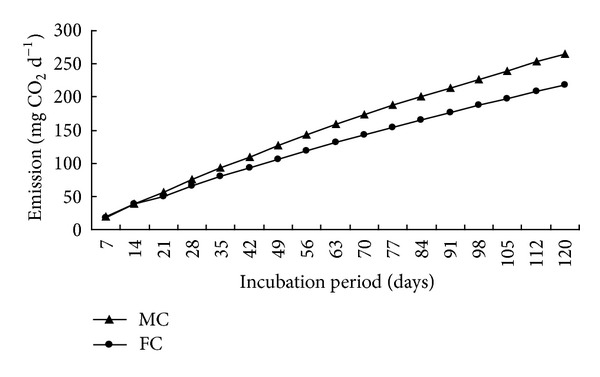
Cumulative CO_2_-C evolution at different water levels.

Effect of organic residues on carbon dioxide emission rate is presented (Figure 3). Carbon dioxide emission results were statistically significant at all the studied durations.

**Figure 5:**
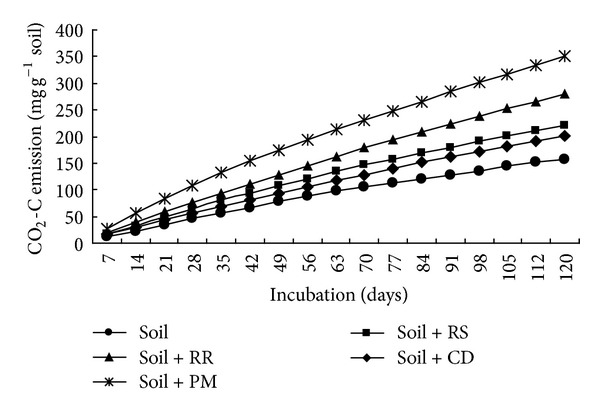
Cumulative CO_2_-C evolution from different organic residues.

Organic residue with mixture of soil was significantly increased carbon dioxide emission over soil alone. Poultry litter produced the highest CO_2_-C evolution from 7 to 120 days after incubation. The second highest CO_2_-C emission was obtained from soil + rice root treated pot. Control treatment performed the lowest CO_2_-C emission during entire period of incubation study. Maximum carbon dioxide emission (0.042 mg d^−1 ^g^−1^ soil) was found in poultry litter mixed with soil at 14 days after incubation, and the lowest carbon dioxide emission (0.017 mg d^−1 ^g^−1^ soil) was found in only soil treated pot. Carbon dioxide emission was decreased with the increase of time. The lowest carbon dioxide emission was found in 120 days after incubation for all the organic residues including control. Similar trend was found in cumulative carbon dioxide emission from different organic residues (Figure 5).

Cumulative CO_2_-C evolution was increased with the increase of time. Mixing of organic residues with soil significantly increased cumulative CO_2_-C. It brought roughly a 121% increase in cumulative CO_2_-C production in poultry litter treated pot compared to control. All the studied organic residues, the cumulative CO_2_-C showed linear trend with significant variation during entire incubation period. In this study, on average about 38% C was mineralized to CO_2_-C in poultry litter treated pots. The percent of carbon mineralized 39.21, 77.13, 42.14, and 114.82 from rice straw, rice root, cow dung, and poultry litter, respectively, over control. The cumulative CO_2_-C emission was 1.39, 1.77, 1.27, and 2.21 times higher in rice straw, rice root, cow dung, and poultry litter, respectively, over control. The CO_2_-C emission trend increased in the order poultry litter > rice root > rice straw > cow dung. The lowest cumulative CO_2_-C evolution was found in 7 days after incubation, and the maximum CO_2_-C evolution was obtained from 120 days after incubation. Total input and output carbon, uncounted carbon and carbon degradation constant rate results are presented (Table 3). Soil and organic residue contributed 0.92 and 0.25 g C per pot, respectively. Higher carbon emission (0.095 g) was found in poultry litter treated pot, and the lowest carbon emission (0.040 g) was observed in soil treated pot. Further, it was observed that after poultry litter decomposition, the amount of soil retained carbon was 1.060 g and evolved C was 0.095 g. Cow dung treated pot produced the lowest CO_2_-C evolution as a results the highest carbon retained was in soil in cow dung treated pot. Maximum apparent carbon balance (1.16 g) was achieved in cow dung treated pot, and the lowest carbon content was found in soil treated pot. Higher uncounted carbon was found in soil + rice root treatment. There were significant differences in the *k* value of organic residues (Table 3). The *k* values of rice straw, rice root, cow dung, poultry litter, and soil alone were 0.003, 0.005, 0.005, 0.008, and 0.008 g per day, respectively. Maximum carbon degradation rate was observed in poultry litter and control treatments, and the lowest carbon degradation rate (0.003 g d^−1^) was found in rice straw treated pot. The second highest carbon degradation rate was obtained in rice root and cow dung treated pot. Rice straw had lower *k* values than those of rice root, cow dung, poultry litter, and soil alone. 

Mixing of organic residues significantly produced more CO_2_-C than control. Incorporation of crop residues provides a source of readily available C and subsequently influences the CO_2_-C emission [9]. The residue type was thought to be an important factor affecting CO_2_-C emission. Decrease in residue mineralization in later stages may indicate that more organic carbon was sequestered in soil or was incorporated into microbial biomass. Similar results were found by [10]. All the studied organic residues, the cumulative CO_2_-C showed linear trend with significant variation during entire incubation period. In agreement with results found that mixing of the maize straw with soil caused almost 40% increases in the cumulative CO_2_-C production than the controls [11]. Elevated rates and cumulative CO_2_-C were observed in poultry litter due to more fine materials than other studied organic residues which favors bacterial activity for the decomposition. Cow dung produced the highest carbon content in soil. Cow dung is a well-decomposed organic material; as a result, it has less amount of labile C for producing CO_2_-C after incorporation in soil. Despite the important role of cow dung to increase carbon content in soil, this material is not affordable for the farmer's because firstly, the number of cattle is decreasing gradually and cow dung is becoming unavailable simultaneously. Secondly, cow dung is not available due to use of fuel purpose. Scientist reported that farmer's used cow dung in field for the crop production, but nowadays it is unavailable for the use of fuel purpose [12]. Annual rice straw production increased from an average of 21.98 × 10^6^ mt per year in the 1978s to 30.84 × 10^6^ mt per year in 2008 in Bangladesh. So, depletion of nutrients and poor organic matter contents of Bangladesh soils can be replenished by incorporation of cost effective and easily available rice straw to increase rice yield and improve soil quality. Rice straw produced the second highest organic carbon in soil due to organic residue decomposition constant rate was lower than other organic residues because rice straw contains high C : N ratio with high amount of nonlabile organic carbon such as lignin. Farmer's believe that burning process of rice straw is inexpensive and effective in removing fungus, while replacing macro- and micronutrients in the soil. However, burning also increases particulate matter in the air which creates environmental pollution, thus, alternative uses are being sought. Incorporation of rice straw provides a very sustainable alternative to conventional burning practices, because it builds soil profile and adds organic matter, nutrients, and beneficial microorganisms to the soil. Rice straw incorporation into the soil at the rate of 5.0 t ha^−1^ did not affect early rice growth stage or rice yield [5]. Mixing of organic residues with soil significantly increased SOC levels compared to controls. Generally, rice straw contains half of carbon, which increases SOC upon mineralization. Rice straw with soil found that the SOC content was enhanced. The significant amount of SOC was obtained in rice straw treated pot [13]. This may be due to nonlabile C and flooding system for unfavorable condition for microbial decomposition. The low microbial activities at higher C : N ratio with nonlabile C content led to slow decomposition of rice straw resulting, thereby, in higher stability of organic carbon in soil. CO_2_-C emission rates were higher in the first week, but after day 14, it declined and these results were more or less consistent with previous works reported that incubated maize straw with soil together with N addition and found that initially rates were higher but declined after 10 days [13]. Enhanced cumulative CO_2_-C production observed in poultry litter treated pot because poultry litter is a fine particle which favors bacterial activity for the decomposition. As a result, poultry litter produced the significant amount of soil organic carbon. Poultry litter at 0, 6, and 12.7 Mg ha^−1^ y^−1^ doses increased significant amounts of carbon sequestration in soil at 0–15 cm depth [14]. So, poultry litter may be a good source of organic residue instead of farmyard manure in Bangladesh in near future because poultry farms are increasing day by day. Rice root was used in this study to compare the efficiency with other organic residues for carbon sequestration in soil. Results revealed that rice root increased carbon content in soil over control. Researchers would get some ideas about the contribution of rice root for carbon sequestration in soil. Degradation constant (*k*) value is good indicator to select effective carbon sequesters in soil and reduce environmental pollution. Different organic residues with different water levels showed different decomposition constant rate due to some reasons. Firstly, decomposition rate depends on C : N ratio of the tested materials. A high C : N ratio containing organic residue slows the rate of residue breakdown because lignin content was higher than other easily decomposable compounds. The rate of decomposition of organic materials composition is in the following order sugars, starches, and proteins > hemicelluloses > cellulose > fats, waxes > lignin. On the other hand, it is especially important where the C : N ratio of organic material is high and thus, decomposition is slowed by a lack of nitrogen. In this regard, decomposition constant rate of rice straw was the lowest among the tested organic materials. This delay may help to reduce nutrient loss and better synchronize nutrient availability and crop demand [15]. Secondly, good aeration is an important factor for the proper activity of microorganisms involved in the decomposition of organic matter. Under anaerobic conditions fungi and actinomycetes are almost suppressed and only a few bacteria take part in anaerobic decomposition. The rate of decomposition is markedly retarded in anaerobic condition. Some researchers claimed that aerobic condition 65 percent of the total organic matter decomposes during six months, while under anaerobic conditions only 47 percent organic matter can be decomposed during the same period. Thirdly, decomposition constant depends on the content of labile and nonlabile C. In this regard, cow dung and poultry litter produced 0.005 and 0.008 g d^−1^ C, respectively. Cow dung produced the lowest CO_2_-C due to nonlabile carbon and sequestrated maximum organic carbon in soil. The highest CO_2_-C was produced in poultry litter treated pots. Poultry litter is a fine material that is why microorganisms decompose it easily. On the other hand, control treatment produced the highest *k* value. From this result, it may be concluded that arable land without vegetation or crops increased CO_2_-C emission through oxidation process for enhancing environmental pollution. Based on these discussions, organic residues along with flooding condition improved soil organic carbon, and reduced environmental pollution.

Interaction results revealed that mixing of organic residues with soil and different levels of water significantly affected rates and cumulative CO_2_-C emission, apparent carbon balance, uncounted carbon and decomposition constant rate of organic residues (Tables 2 and 3). MC × soil increased 13% emission carbon over MC × soil. FC × soil + poultry litter treated pot produced 100% more CO_2_-C over FC × Soil. Organic residues in combination with water level produced the maximum CO_2_-C rate and cumulative CO_2_-C over soil alone with water level. Maximum residue organic carbon content (1.11 g) was observed in FC × soil + rice straw and FC × soil + cow dung treated pot. The lowest residue organic carbon content was found in FC × soil. Lowest uncounted carbon was found in FC × soil. Maximum *k* value was found in moistened condition. Based on these results, moistened condition with different organic residues including control produced the higher CO_2_-C emission than in flooding condition with different organic residues including control.

## Figures and Tables

**Table 1:** Characteristics of organic residues and soil.

Parameters	%C	%N	%P	%K	C : N ratio
Soil	0.92	0.08	0.0098	0.0025	11.5
Rice straw	48.90	0.63	0.08	2.35	77.61
Rice root	42.20	0.40	0.29	0.34	105.5
Cow dung	17.43	1.04	0.82	0.68	16.75
Poultry litter	47.41	1.00	0.69	0.95	47.41

**Table 2:** Interaction effect of organic residues and water levels on carbon dioxide evolution.

Treatment	Incubation period (days)																
	7	14	21	28	35	42	49	56	63	70	77	84	91	98	105	112	120
	CO_2_-C emission (mg g^−1^ soil)																
Carbon emission																	
MC × Soil	0.016	0.015	0.015	0.015	0.015	0.014	0.013	0.013	0.013	0.013	0.012	0.012	0.012	0.012	0.012	0.016	0.012
MC × Soil + RS	0.024	0.024	0.024	0.024	0.024	0.023	0.023	0.023	0.022	0.022	0.016	0.016	0.016	0.016	0.016	0.016	0.016
MC × Soil + RR	0.030	0.030	0.030	0.030	0.029	0.027	0.028	0.029	0.029	0.029	0.023	0.022	0.022	0.022	0.022	0.022	0.022
MC × Soil + CD	0.022	0.018	0.018	0.018	0.018	0.017	0.018	0.017	0.017	0.017	0.017	0.014	0.014	0.015	0.014	0.014	0.015
MC × Soil + PM	0.042	0.050	0.047	0.043	0.041	0.037	0.036	0.034	0.034	0.030	0.030	0.030	0.030	0.030	0.029	0.029	0.028
FC × Soil	0.018	0.017	0.017	0.017	0.016	0.016	0.097	0.016	0.016	0.010	0.010	0.010	0.010	0.010	0.010	0.010	0.009
FC × Soil + RS	0.023	0.023	0.021	0.023	0.022	0.016	0.016	0.021	0.015	0.015	0.015	0.015	0.015	0.015	0.012	0.012	0.012
FC × Soil + RR	0.026	0.025	0.024	0.021	0.022	0.021	0.021	0.017	0.021	0.021	0.019	0.019	0.019	0.019	0.019	0.019	0.019
FC × Soil + CD	0.025	0.020	0.020	0.020	0.018	0.021	0.017	0.021	0.017	0.017	0.017	0.017	0.014	0.014	0.014	0.014	0.014
FC × Soil + PM	0.036	0.034	0.030	0.030	0.029	0.021	0.021	0.027	0.021	0.021	0.021	0.021	0.021	0.019	0.018	0.018	0.018
LSD_0.05_	NS	0.023	0.022	0.022	0.070	0.023	NS	0.022	0.023	0.007	0.023	0.022	NS	0.022	NS	NS	0.007
Cumulative carbon emission																	
MC × Soil	11.32	22.17	32.96	43.80	54.27	64.28	73.64	82.63	91.72	100.79	108.87	116.97	125.04	133.15	141.21	149.32	157.56
MC × Soil + RS	16.99	33.77	50.51	67.31	83.79	99.83	116.21	132.29	147.39	162.44	173.46	184.51	195.60	206.79	217.91	229.50	240.26
MC × Soil + RR	21.27	42.05	62.78	83.62	104.08	123.17	142.49	162.58	182.68	202.78	218.85	233.94	249.04	264.15	279.26	294.37	309.59
MC × Soil + CD	15.34	28.24	41.08	53.94	66.25	78.49	90.85	102.95	115.08	127.17	139.26	149.36	159.48	169.65	179.76	189.85	200.08
MC × Soil + PM	29.29	64.10	96.90	126.76	155.26	181.29	206.63	230.72	254.83	275.89	296.96	318.01	339.12	360.29	380.41	400.51	419.77
FC × Soil	12.38	24.31	36.12	47.90	59.43	70.51	81.59	92.59	103.69	110.80	117.90	124.98	132.08	138.95	145.94	152.92	158.90
FC × Soil + RS	16.24	32.10	46.87	62.62	78.14	89.10	99.96	110.58	121.20	131.82	142.44	153.05	163.69	174.37	182.97	191.58	200.29
FC × Soil + RR	18.33	36.17	52.94	67.72	83.24	98.16	112.95	127.54	142.05	156.61	170.15	183.69	197.24	210.79	224.34	237.88	251.52
FC × Soil + CD	17.32	31.23	45.00	58.79	71.35	83.24	94.99	106.55	118.13	129.69	141.25	152.81	162.37	171.93	181.47	191.03	200.65
FC × Soil + PM	25.32	49.20	70.06	90.84	111.35	126.27	141.07	155.67	170.27	184.85	199.43	214.02	228.60	242.19	254.77	267.36	280.04
LSD_0.05_	NS	7.24	6.71	5.45	6.49	5.22	7.11	4.79	8.27	7.07	11.20	13.58	8.15	7.72	8.13	9.43	1.01

**Table 3:** Carbon balance from different organic residues in 100 g soil.

Treatment	Carbon input (g)			Carbon output (g)			Uncounted C (g)	Degradation rate: *k* (g d^−1^)
	Soil carbon	Residues carbon	Total carbon	Emission carbon	Residual carbon	Apparent C balance		
Water levels (W)								
MC	0.92	0.20	1.12	0.072	1.012	1.084	0.036	0.007
FC	0.92	0.20	1.12	0.058	1.032	1.090	0.030	0.005

Organic residues (OR)								
Soil	0.92	0.00	0.92	0.040	0.870	0.911	0.010	0.008
Soil + RS	0.92	0.25	1.17	0.060	1.050	1.109	0.061	0.003
Soil + RR	0.92	0.25	1.17	0.076	1.025	1.101	0.069	0.005
Soil + CD	0.92	0.25	1.17	0.054	1.105	1.160	0.011	0.005
Soil + PM	0.92	0.25	1.17	0.095	1.060	1.155	0.015	0.008

Interaction (W × OR)								
MC × soil	0.92	0.00	0.92	0.043	0.870	0.913	0.007	0.008
MC × soil + RS	0.92	0.25	1.17	0.065	0.990	1.053	0.117	0.001
MC × soil + RR	0.92	0.25	1.17	0.084	1.060	1.144	0.026	0.008
MC × soil + CD	0.92	0.25	1.17	0.054	1.100	1.154	0.016	0.005
MC × soil + PM	0.92	0.25	1.17	0.114	1.040	1.154	0.016	0.010
FC × soil	0.92	0.00	0.92	0.038	0.870	0.908	0.012	0.008
FC × soil + RS	0.92	0.25	1.17	0.055	1.110	1.165	0.005	0.004
FC × soil + RR	0.92	0.25	1.17	0.068	0.990	1.058	0.112	0.001
FC × soil + CD	0.92	0.25	1.17	0.055	1.110	1.165	0.005	0.004
FC × soil + PM	0.92	0.25	1.17	0.076	1.080	1.156	0.014	0.007
LSD_0.05_								
W	NS	NS	NS	0.013	NS	NS	0.005	0.002
OR	NS	0.016	0.072	0.016	0.143	0.113	0.016	0.005
W × OR	NS	NS	NS	NS	NS	NS	0.023	0.022
